# Estimating Cognitive Reserve in Healthy Adults Using the Cognitive Reserve Scale

**DOI:** 10.1371/journal.pone.0102632

**Published:** 2014-07-22

**Authors:** Irene León, Juan García-García, Lola Roldán-Tapia

**Affiliations:** Department of Psychology, University of Almeria, Almeria, Spain; University Of São Paulo, Brazil

## Abstract

The concept of cognitive reserve emerged from observed disparities between brain pathology and clinical symptoms. It may explain better neuropsychological performance in healthy individuals. The objectives of this study were to measure reserve in healthy subjects using a new Cognitive Reserve Scale (CRS), analyze the internal consistency of the CRS, and analyze validity evidence. A total of 117 healthy individuals were divided into two groups: 87 adults (aged 18–64 years) and 30 elderly adults (≥65 years). All subjects completed the CRS and a comprehensive neuropsychological battery. The internal consistency of the scale was satisfactory (α = 0.77). No significant differences were observed between genders (t = 0.51, p = 0.611), and age was corrected by averaging the CRS score. The study of validity evidence showed that education affected the CRS (t = −2.98, p = 0.004, partial h^2^ = 0.07) and there was no significant relationship between the CRS and IQ (r = 0.09, p = 0.33). Occupational attainment and the CRS were not related (F_2,116_ = 0.11, p = 0.898). In line with previous studies on reserve, heterogeneity was observed in the analyses of relationships between the CRS and cognitive performance. There were significant relationships between CRS score and the Verbal Learning Spanish–Complutense Test last trial (r = 0.24, p = 0.009), sum (r = 0.32, p = 0.000), short-term (r = 0.29, p = 0.002) and long-term memory (r = 0.22, p = 0.018), Matrix Reasoning subtest (r = 0.20, p = 0.027) and Block Design subtest (r = 0.20, p = 0.029). No other neuropsychological variables correlated with the CRS (p>0.05). The CRS is a reliable instrument that reflects the frequency of participation in brain-stimulating activities across the lifetime. The associations between the CRS and education and neuropsychological performance support validity evidence.

## Introduction

The concept of reserve emerged from the lack of correlation between brain pathology and clinical symptoms. The reserve theory postulates that individuals with a greater reserve will cope with brain damage more successfully than those with low levels of reserve [1]. Reserve provides protection that contributes toward delaying age-related changes and clinical symptoms related to underlying neuropathological processes, such as Alzheimer's disease [2]–[6]. However, this protection does not prevent some symptoms appearing in the long term [7].

In recent decades, the number of studies on reserve has increased notably [8]–[15]. The interest that reserve initially aroused in the field of Alzheimer's disease [1], [16] has spread to a wide variety of studies related to healthy aging, multiple sclerosis, mild cognitive impairment, heart failure and schizophrenia [17]–[21].

The reserve theory distinguishes between two models: cerebral reserve and cognitive reserve (CR). The *passive* or *cerebral reserve model* postulates that brain pathology can accumulate to a critical threshold at which symptoms appear [22]. The *active* or *cognitive reserve model*, from the perspective of cerebral plasticity, postulates that the brain has the capacity to cope with damage through compensatory mechanisms, or through flexible and adaptive networks [1]. The active model has repercussions for healthy people. Individuals with a higher CR may have more efficient networks, which would allow them to achieve better performance on cognitive tasks than individuals with a lower CR [1], [23]. Some authors have suggested studying the passive and active models in combination, because they complement each other [13], [24]–[27].

In the passive model, cerebral reserve is mainly quantified by brain size or intracranial volume and by head circumference [26]–[28]. In contrast, in the active model, years of education and premorbid intelligence quotient (IQ) are used as CR proxies. IQ is usually measured by the Vocabulary subtest of the Wechsler Adult Intelligence Scale [13], [23], [29]–[31] or by reading tests such as the National Adult Reading Test [30], [32]–[35]. Educational attainment either takes into account the total number of years of education or is classified into high and low educational levels [11], [17], [28], [36], [37]. Occupational attainment is another commonly used proxy of CR [8], [14], [38].

CR may be attained through an active cognitive lifestyle, which involves engaging in cognitively stimulating activities. Participation in intellectual, social, physical or leisure activities contributes toward delaying or attenuating symptoms related to brain damage and reduces the risk of dementia [2], [39]–[43]. The methodological instruments used in CR studies, such as the Cognitive Activities Scale [40], Activities Scale [9] or Lifetime of Experiences Questionnaire [42], have influenced the development of the Cognitive Reserve Questionnaire [44], Cognitive Reserve Index Questionnaire [45] and Cognitive Reserve Scale (CRS) [46]. In 2013, a new measure of premorbid cognitive abilities in subjects with low educational attainment, the Premorbid Cognitive Abilities Scale, was suggested as another proxy of CR [47].

Psychometric analyses of questionnaires and scales on CR, along with assessment of their reliability and validity evidence [40], [42], [45], [46], have provided evidence for their application. Significant statistical correlations have been observed between these CR proxies and a decline in cognitive domains, which reflects the relevance of such research studies, although they do not seem to follow any clear or consistent pattern [40], [42]. Indeed, the theory of CR implies that this construct enables coping with challenges in general, and not only on a specific cognitive domain [23]. In addition, reserve is a hypothetical construct that cannot be measured directly; therefore, its measurement presents a challenge to the scientific community [28], [48].

This study was based on the active or CR model and its influence on healthy individuals [1], [23]. The CRS is a new instrument that can be used to obtain a measure of reserve focused on accounts of participation in stimulating activities throughout life. Based on previous studies, it was hypothesized that: (i) participants with more years of education would have higher CRS scores; (ii) occupational attainment would influence the CRS score; (iii) IQ and the CRS would not be associated; and (iv) relationships between neuropsychological scores and the CRS score would be heterogeneous, with significant relations expected between CRS score and memory tasks.

The overall aim was to study the psychometric properties of the CRS and support CR studies based on cognitively stimulating activities across the lifetime.

## Materials and Methods

### Ethics statement

This study was approved by the Ethics Committee of the University of Almeria, and conducted in compliance with the Declaration of Helsinki and Spanish legislation on personal data protection. All subjects were volunteers and they provided written consent.

### Subjects

Participants were recruited from social clubs, social centers, entertainment centers and the University of Almeria. The sample (n = 154) was split into two age groups according to the study design: adults (aged 36–64 years) and elderly adults (≥65 years). The traditional Spanish retirement age (65 years) determined the classification of the two age groups in this study. Individuals were excluded from the analysis if they had a history of psychiatric or neurological illness, drug consumption or head injury, or if they were non-native Spanish. Elderly adults (≥65 years) were also excluded if they gained a score of 27 or lower on the Spanish adaptation of the Mini-Mental State Examination [49], Mini-Examen Cognoscitivo [50]. Following these criteria, and after removing subjects who decided not to complete the study, 37 participants (24%) were excluded. This affected the gender distribution in both age groups. The remaining sample of 117 subjects comprised 87 adults (mean ± SD age  = 48.76±0.758 years), 54 (62.1%) of whom were women, and 30 elderly adults (age 72.9±1.102 years), 22 (73.3%) of whom were women. Table 1 shows the sociodemographic characteristics of the participants.

**Table 1:** Sociodemographic characteristics of the study participants (N = 117).

	n	%
**Gender**		
Male	41	35.0
Female	76	65.0
**Age group**		
Adults (36–64 years)	87	74.4
Elderly adults (≥65 years)	30	25.6
**Educational attainment**		
High (>8 years)	29	24.8
Low (≤8 years)	88	75.2
**Occupational attainment**		
High	19	16.2
Medium	36	30.8
Low	62	53.0

### Educational and occupational attainment

Educational attainment was stratified into high level (>8 years of education) and low level (≤8 years of education) [2]. Regarding occupational attainment, each subject's primary occupation was recorded using the Spanish National Classification of Occupations (Clasificación Nacional de Ocupaciones) [51] and stratified into high, medium and low levels following similar classifications used in previous studies on CR [3], [24]. High level occupations included managers, scientific and intellectual technicians and professionals; medium level included clerks, accountants and related professionals, and professionals in the armed forces; and low level included sales agents and customer service employees, skilled workers in the agricultural, forestry and fishery industry, workers in crafts and related trades, plant and machine operators, elementary occupations and home-makers (Table 1).

### Cognitive Reserve Scale

**Figure 1:**
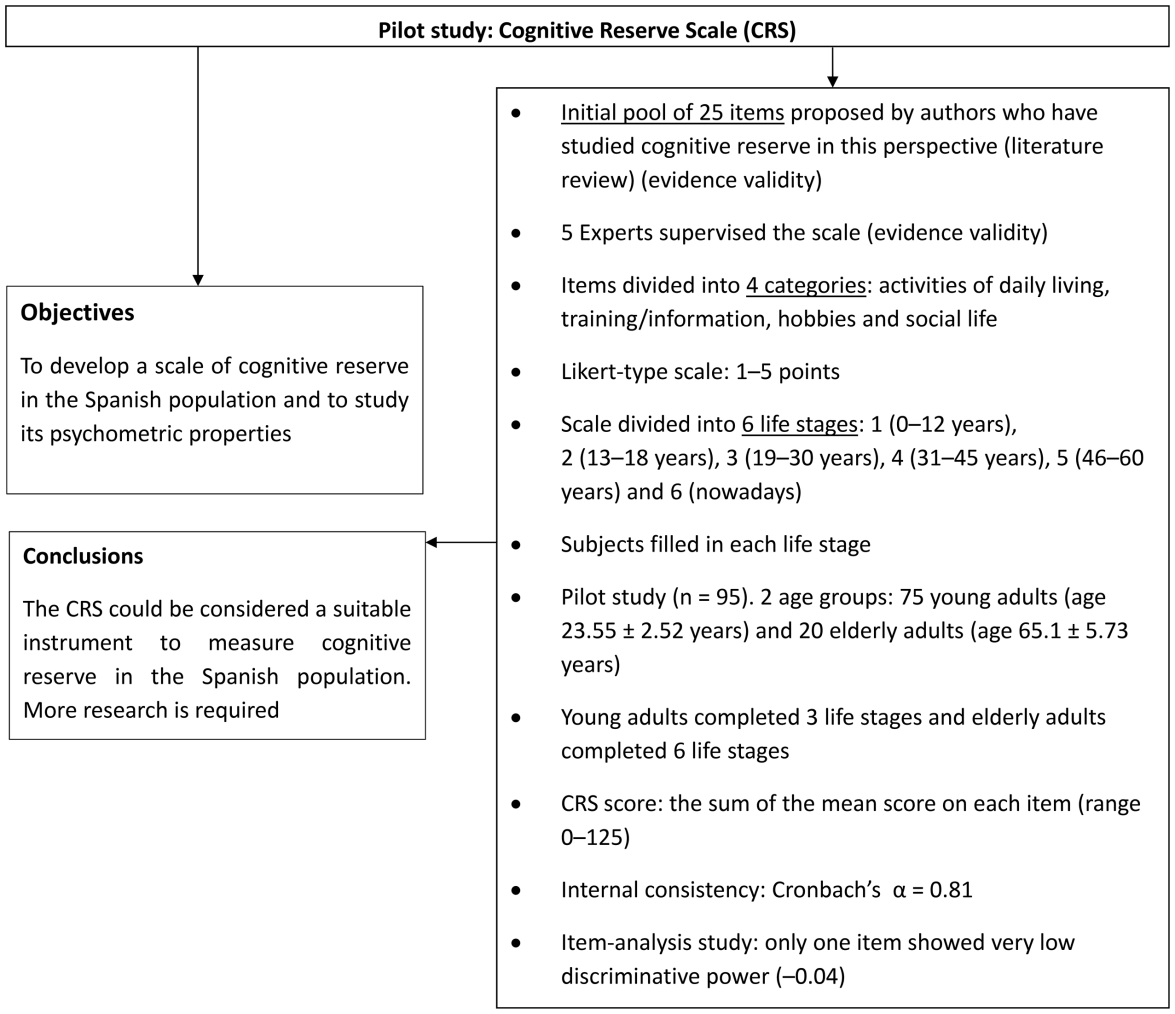
Flow diagram of the pilot study of the Cognitive Reserve Scale (CRS) (León et al., 2011) [46].

**Figure 2:**
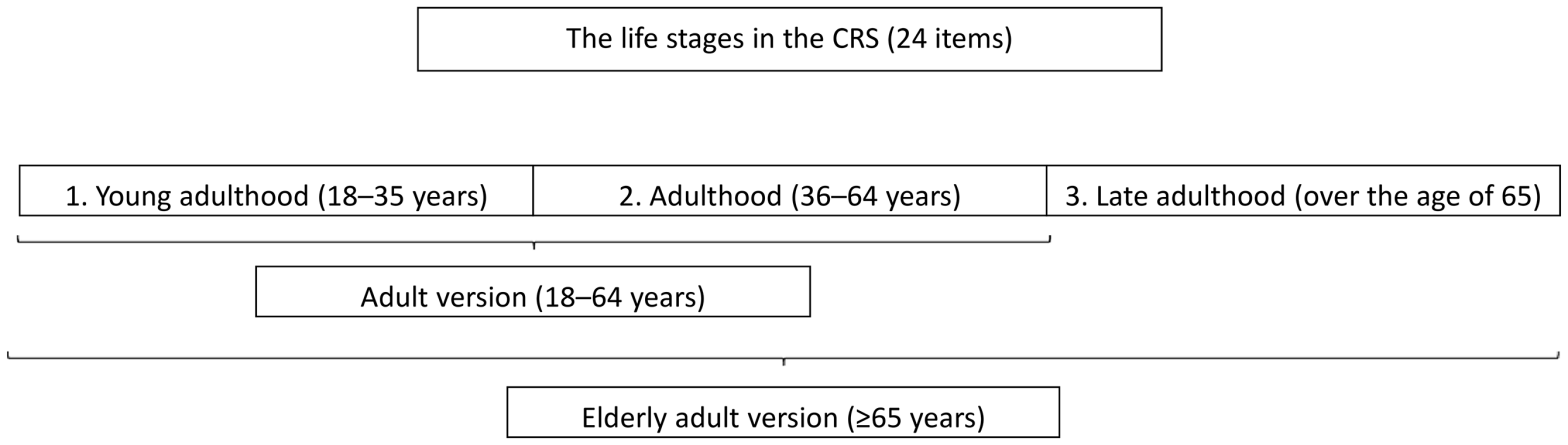
The three life stages included in the Cognitive Reserve Scale (CRS).

The CRS is a new test that measures participation in cognitively stimulating activities throughout a person's lifetime [46]. A pilot study on the CRS is summarized in Figure 1. There were 24 items in total and subjects completed each one several times, according to their age, because the CRS was divided into three different life stages (Figure 2).

The specific age cut-offs for the life stages were established according to the results of previous studies on CR and stimulating cognitive activities throughout the lifetime [42], [46]. Items included a diverse variety of activities such as reading, playing a musical instrument, collecting things, practicing other language or dialects, traveling or taking part in sport. These activities have been proposed by authors studying CR from this perspective. The CRS was divided into four categories: activities of daily living, training–information, hobbies and social life. Table 2 shows examples of translated items in each category of the scale. The CRS is available upon request from the authors.

**Table 2:** Examples of translated items on the Cognitive Reserve Scale (CRS).

Category	Example items
**Activities of daily living**	Controlling my own life (e.g. what to wear each day, hotel bookings, doctor's appointments)
	Controlling financial matters at home (e.g. bills, mortgage)
**Training/information**	Taking a course (e.g. language, Internet use)
	Speaking a non-native language or dialect
**Hobbies**	Reading (e.g. newspapers, magazines, books)
	Playing games (e.g. crosswords, sudoku, cards, draughts, chess)
	Writing for pleasure (e.g. letters, personal diary)
	Listening to music or watching television
	Playing a musical instrument (e.g. guitar, flute)
	Collecting things (e.g. stamps, coins)
**Social life**	Visiting relatives, friends, neighbors, etc.
	Volunteering, going to church, etc.

Subjects completed each item several times according to their age (Figure 2). A Likert-type scale of 0–4 points was used and the total CRS score was the sum of the mean score on each item (24 items). It is important to note that this mean score on each item corrected for the possible effect of the additional period, called late adulthood, for elderly adults. As a result, the CRS gave scores varying from 0 to 96, with higher CRS scores indicating more frequent participation.

### Neuropsychological assessment

Six cognitive domains from different subtests of the Wechsler Adult Intelligence Scale [29] and other neuropsychological tests were evaluated. Each cognitive domain included a minimum of two tests: (1) memory was assessed with the Verbal Learning Spanish–Complutense Test (TAVEC) [52] and Rey–Osterrieth Complex Figure (ROCF) [53]–[55]; (2) working memory was tested with the Digit Span subtest (backward) [29], [56], [57] and Corsi's Block-Tapping test (backward) [56], [57]; (3) attention was evaluated with the Digit Span subtest (forward) [29], [56], [57], Corsi's Block-Tapping test (forward) [56], [57] and the Stroop test [58]–[60]; (4) executive functions were assessed with the FAS test and Animals [61]–[63] and the Matrix Reasoning subtest [29]; (5) visuoconstruction and visuoperception were tested with the Block Design subtest [29] and the copy of ROCF [53]–[55]; and (6) processing speed (seconds) was evaluated with the copy of ROCF and the Trail Making Test (parts A and B) [53]–[57]. In addition, the Vocabulary subtest was applied to obtain an IQ score [29]. The direct scores of all neuropsychological tests were converted to standard scores adjusted for age and educational level (scale scores, z-scores or percentile scores) following normative studies in the Spanish population. The exception was the FAS test, which followed a normative study in an English population which was stratified for age and years of education [61].

Elderly adults (≥65 years) also completed the Spanish version of Mini-Mental State [49], Mini-Examen Cognoscitivo [50], which includes items about abstract reasoning and working memory. A trained psychologist performed the assessments.

### Statistical analysis

A descriptive analysis of the distribution of total CRS scores was carried out for the whole healthy sample. The internal coherence of the scale was estimated using Cronbach's alpha test. The t-test was used to study the effects of gender and age on the CRS score. The possible effect of age was corrected by averaging the CRS score, and the t-test was also used to support the lack of effect of age on the score. To verify whether educational attainment (low/high) affected the CRS score, the t-test was used. One-way analysis of variance (ANOVA) was used to examine the influence of occupational attainment on the CRS. Relationships between the total CRS score and IQ, and the total CRS score and cognitive tests, were evaluated with Pearson's correlations. Analyses were carried out with the statistical package IBM-SPSS (version 20.0 for Windows). Results with p<0.05 were considered statistically significant.

## Results

### Cognitive Reserve Scale scores

**Figure 3:**
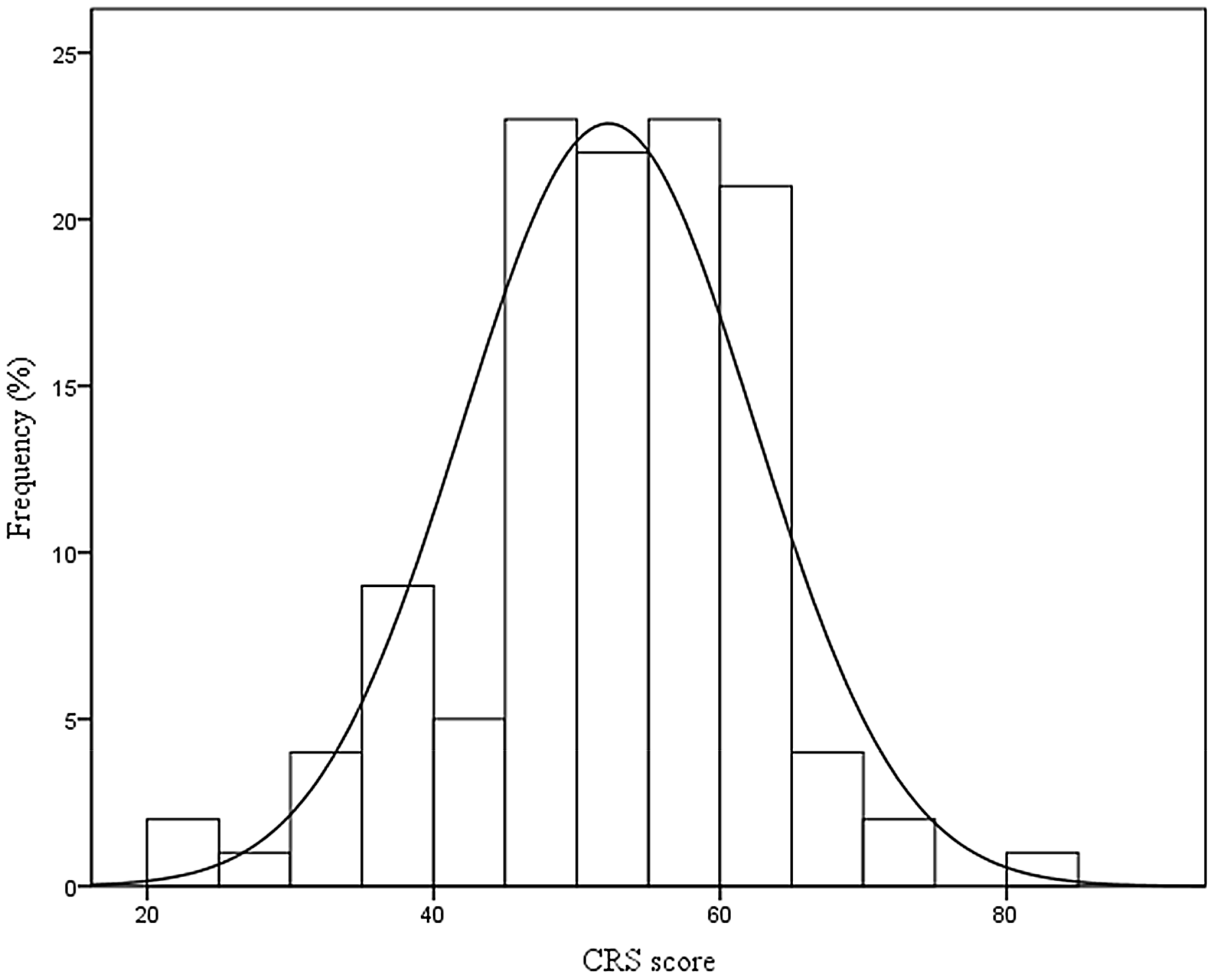
Distribution of Cognitive Reserve Scale (CRS) scores for all participants.

A composite measure of frequency of activities on the CRS (24 items) across the three life stages (young adulthood, adulthood and late adulthood) was formed by computing the sum of the mean score on each item (mean ± SD  = 52.22±10.30). Subjects completed each item several times according to their age (Figure 2). There were no missing values on any items of the CRS. The CRS generated scores varying from 0 to 96, with higher scores indicating more frequent participation. Thus, having a more active lifestyle was interpreted as having more CR. The Kolmogorov–Smirnov test gave a non-significant result (p = 0.201), suggesting normality of the distribution. The distribution of the CRS scores and the percentile breakdown for all participants are presented in Figure 3 and Table 3, respectively. The analysis revealed that there were no significant differences in the CRS score between men and women (t = 0.51, p = 0.611) and that age (adults versus elderly adults) did not affect the CRS (t = 0.87, p = 0.384).

**Table 3:** Percentiles for the total Cognitive Reserve Scale (CRS) score.

Percentile	Total CRS score
95	67.03
90	63.50
75	59.50
50	53.00
25	46.58
10	37.00
5	33.97

### Internal consistency

The total score on the CRS showed high internal consistency (Cronbach's α = 0.77), supporting the idea that a composite measure could adequately summarize the frequency of participation in activities throughout the lifetime [41]. This alpha value was similar to that obtained in the pilot study (α = 0.81).

### Validity evidence

Relationships between the total CRS score and other CR proxies commonly used in studies on CR, such as years of education, occupational attainment and IQ score, were assessed. It was hypothesized that education and occupational attainment could be related to the CRS score, but no relation to IQ was expected [45]. The results showed that educational attainment affected the CRS scores (t = −2.98, p = 0.004, partial h^2^ = 0.07), whereas occupational attainment was not related to the CRS (F_2,116_ = 0.11, p = 0.898). As expected, no significant relationship was found between the total CRS score and IQ (Vocabulary subtest) (r = 0.09, p = 0.33). The distribution of participants according to educational attainment and occupational attainment is presented in Table 1. The observed effect size was medium (partial h^2^≥0.05).

Since the literature shows heterogeneity with regard to relationships among proxies of CR based on activities across the lifespan and cognitive results, it was hypothesized that the relationships between the CRS and the neuropsychological variables would show heterogeneity and a lack of negative relationships. It can be observed in Table 4 that: (i) there were positive correlations between the CRS score and TAVEC last trial, TAVEC sum, and TAVEC short-term and long-term memory (verbal memory); (ii) there was a positive correlation between the CRS score and the Matrix Reasoning subtest (executive functions); (iii) the CRS score and the Block Design subtest were also significantly associated (visuoconstruction–visuoperception); (iv) the CRS score was not correlated with any test of working memory, attention and processing speed; and (v) no significant relationships were demonstrated between the CRS score and TAVEC first trial, TAVEC recognition, ROCF, FAS and Animals. As expected, all of the significant correlations were positive, and the sizes of the correlations were medium to low.

**Table 4:** Correlations between the Cognitive Reserve Scale (CRS) score and each cognitive variable classified into six cognitive domains.

Cognitive domain		CRS score			
	n	Mean	SD	r	p
**Working memory**					
Digit Span (backward)	117	10.68	2.53	0.077	0.409
Corsi's Block-Tapping (backward)	117	15.19	2.66	−0.071	0.450
**Memory**					
TAVEC – first trial	117	0.02	0.95	0.102	0.273
TAVEC – last trial	117	0.67	0.98	0.241	0.009
TAVEC sum	117	0.46	0.88	0.320	0.000
TAVEC short-term	117	0.45	0.98	0.286	0.002
TAVEC long-term	117	0.39	1.13	0.219	0.018
TAVEC recognition	117	0.20	0.93	0.098	0.291
ROCF short-term	116	9.36	4.93	0.066	0.485
ROCF long-term	116	8.78	2.91	0.060	0.525
**Attention**					
Digit Span (forward)	117	9.97	2.71	−0.035	0.708
Corsi's Block-Tapping (forward)	117	14.6	2.93	−0.109	0.244
Stroop: word–color score	117	9.76	2.75	0.135	0.147
**Executive functions**					
FAS sum	117	47.66	28.31	0.095	0.309
Animals	117	10.56	2.85	0.145	0.118
Matrix Reasoning	117	11.96	2.94	0.204	0.027
**Visuoconstruction-visuoperception**					
Block Design	117	11.21	2.80	0.202	0.029
ROCF copy	116	7.23	2.64	−0.067	0.477
**Processing speed**					
Time to copy ROCF (s)	116	9.13	2.59	0.018	0.847
TMT-A	117	10.68	2.59	−0.091	0.328
TMT-B	116	9.28	2.63	0.091	0.330

TAVEC: Test de Aprendizaje Verbal España–Complutense (Verbal Learning Spanish–Complutense Test); ROCF: Rey–Osterrieth Complex Figure; TMT: Trail Making Test.

## Discussion

The CRS is a new CR proxy based on a person's participation in cognitively stimulating activities throughout his or her lifetime [46]. The psychometric results in the current study suggest that the CRS is an adequate tool for assessing CR in the Spanish population. With regard to sociodemographic characteristics, it was observed that the CRS score was not affected by gender, and the possible effect of age was corrected by averaging the CRS score. Thus, age did not influence the CRS score and was controlled for so that it did not bias the results.

Regarding the study of validity evidence, it should be noted that the development of the CRS was based on the frequency of participation in brain-stimulating activities across the lifespan and that different analyses were performed to study the relationships between the CRS score and other frequent proxies of CR (education, occupational attainment and IQ), as well as the associations between the CRS and cognitive performance.

Differences in CRS were found between participants who had attained a high level of education and those who had not. The finding that educational attainment has an effect on the CRS score was expected. For example, Wilson et al. [40] found that a composite measure of cognitive activity frequency correlated with education, and Nucci et al. [45] reported a correlation between leisure time and education subscores on the Cognitive Reserve Index Questionnaire. However, in both studies, the values of the correlations were low (r≤0.30) and different measures of education were applied. Likewise, it was hypothesized that occupational attainment (high, medium and low) would affect the CRS score, although no relationship was found between these two variables. Occupation may provide an indication of experiences, but it is unrealistic to expect a connection with the activities included in the CRS (activities of daily living, training–information, hobbies and social life), as was hypothesized. Furthermore, the association between cognitively stimulating activities across the lifetime and occupational attainment has not been studied in depth.

In this investigation, as in many previous CR studies [20], [30], [64], verbal IQ was estimated using the Vocabulary subtest [29]. This measure of IQ was not included as part of the total CRS score [45], [46]. Although IQ is a very common measure of CR, the approach taken in this study was based on the construct of CR as the variety of brain-stimulating activities that people took part in during their lifetime. Therefore, the two measures, IQ and CRS score, did not share the same definition of CR and it was reasonable to expect a lack of association between these variables. Thus, further psychometric research is needed on the operational and relational definitions of CR, and the properties of the instruments used to measure it.

In agreement with previous CR studies focusing on active cognitive lifestyles, heterogeneity was observed in the relationships between the CRS and cognitive performance. As hypothesized, there were significant relationships between the CRS score and memory tests, and specifically verbal memory tests. However, no associations were found with non-verbal memory tasks. In addition, according to the hypothesis, significant correlations were found between the CRS and abstract tests such as the Matrix Reasoning and Block Design subtests. No other neuropsychological variable was related to the CRS score.

These results are in line with the active model of CR, which postulates that high CR allows subjects to solve more successfully cognitive tasks in general, but not necessarily in a specific cognitive domain [23]. High levels of reserve are associated with more flexible and effective networks that promote the capacity to cope with brain pathology and lead to better performance in healthy subjects [1], [65].

Previous research that focused on CR as the frequency of participation in cognitively stimulating activities throughout life showed diverse associations between reserve and neuropsychological performance. Valenzuela and Sachdev [42] found that, after a follow-up period of 18 months, the relationship between the total score on the Lifetime of Experiences Questionnaire and neuropsychological change was only significant for the attentional domain (r = 0.32, p = 0.01). Another longitudinal study revealed that cognitive activities were associated with a decrease in cognitive deficit in general, working memory and perceptual speed [40]. Ramí et al. found that the Cognitive Reserve Questionnaire was associated mainly with executive tasks in subjects with Alzheimer's disease and healthy subjects [44]. Sánchez et al. [66] developed a model that included the frequency of participation in a variety of activities and demonstrated that people with high CR have better neuropsychological performance than those with low CR.

It is important to note that, in these studies, (i) the cognitive domains did not include the same tests, (ii) different frequency accounts of cognitive activities were used, (iii) different numbers of activities were considered, and (iv) different life stages were considered. Thus, there is a need to continue carrying out studies in this vein [45], [47]. The present study follows this line of research. The CRS was influenced by previous studies based on the frequency of participation in leisure activities [9], [40], in which different periods of life were considered [42]: young adulthood (18–35 years), adulthood (36–64 years) and late adulthood (over the age of 65 years).

Proxies of CR that may be strongly influenced by culture or education (reading level, IQ or years of education) could explain their associations with specific cognitive tasks [21], [35]. However, these CR proxies also correlate with abstract and reasoning tasks that are initially less influenced by education [19], [21]. Zahodne et al. [67], using a large sample (1014 participants), demonstrated that education influenced cognitive performance. The authors suggested that education was associated with different cognitive domains (processing speed, working memory, verbal fluency and verbal episodic memory); in contrast, education had no influence on cognitive decline with age [68]. Thus, some observations must be stressed: (i) heterogeneous results were observed in CR studies; (ii) CR is a hypothetical construct that is not directly measurable; and (iii) the most appropriate CR proxy has not been defined [28].

The well-known benefits of an enriched environment may be comparable with those gained from an active lifestyle. These benefits could contribute toward delaying the risk of dementia or cognitive impairment associated with aging [69]–[71], and may promote brain changes through neuroplasticity and neuroprotection [72], [73]. Lee [74] suggested that to comprehend CR complex networks, studies need to include biomarkers and neuroimaging techniques. For example, Yaffe et al. [75] demonstrated that CR modifies the association between beta-amyloid in the blood and cognitive impairment in elderly people. In short, environmental and genetic factors could contribute to the development of neurodegenerative disorders, such as Alzheimer's disease [76], and it is apparent that studies on CR can be very complex.

Some limitations of this study should be noted. First, the sample size was small and recruitment to the study was not randomized; therefore, further research should include more participants and control recruitment to confirm the present results. Second, a longitudinal study could have gathered information to complete analyses on both the CRS score and potential cognitive change. Finally, although diverse cognitive tasks were included in the cognitive domains, other tasks could be considered to analyze the relation between CRS scores and neuropsychological performance.

In conclusion, the psychometric analyses in this study suggest that the CRS is an adequate test for assessing CR in the Spanish population. Educational attainment influenced the CRS score, and significant relationships were found between the CRS and the memory and abstract reasoning domains. The CRS could be a powerful tool for use in clinical contexts.
